# Construction of functional surfaces for dental implants to enhance osseointegration

**DOI:** 10.3389/fbioe.2023.1320307

**Published:** 2023-11-14

**Authors:** Zhenshi Wang, Jiaolong Wang, Runfa Wu, Junchao Wei

**Affiliations:** ^1^ School of Stomatology, Nanchang University, Nanchang, China; ^2^ Jiangxi Province Key Laboratory of Oral Biomedicine, Nanchang, China; ^3^ Jiangxi Province Clinical Research Center for Oral Disease, Nanchang, China; ^4^ College of Chemistry and Chemical Engineering, Nanchang University, Nanchang, China

**Keywords:** dental implants, osseointegration, surface modification, coating, biomimetic, bone regeneration

## Abstract

Dental implants have been extensively used in patients with defects or loss of dentition. However, the loss or failure of dental implants is still a critical problem in clinic. Therefore, many methods have been designed to enhance the osseointegration between the implants and native bone. Herein, the challenge and healing process of dental implant operation will be briefly introduced. Then, various surface modification methods and emerging biomaterials used to tune the properties of dental implants will be summarized comprehensively.

## 1 Introduction

Due to the tumor recession, injuries, periodontitis, and the aging of population, the defect or loss of dentition has been a common problem in the world, which has greatly affected the daily life of vast numbers of patients (Gulati et al., 2023). Over the past 50 years, implant dentistry has evolved into a highly reliable choice for replacing lost teeth (Buser et al., 2017). Up to now, implanted teeth have been recognized as an ideal alternative of permanent teeth, so dental implants have aroused great attention worldwide.

Dental implants have obtained favorable clinical results and profoundly altered patients’ lives. The global dental market is growing and is expected to be 13.1 billion dollars by 2023 (Alghamdi and Jansen, 2020). Despite dental implants having a 95% estimated 10-year survival (Fischer and Stenberg, 2012), implant failure or loss is still a tough problem. Many studies have demonstrated that bacterial infections, biomechanical mismatch, smoking, aging, and systemic disorders, such as diabetes, osteoporosis, obesity, and the use of drugs, are all factors that can hamper bone regeneration and thus result in the failure of dental implants (Tomasi and Derks, 2022). When the reasons are referred from a more direct or exact point, “failure of osseointegration” is the answer (Alghamdi and Jansen, 2020). It was reported that both the early and late implant loss were related to a failure in osseointegration (Tomasi and Derks, 2022). Thus, optimizing and modifying dental implants to obtain a better osseointegration is still an urgent need.

**FIGURE 1:**
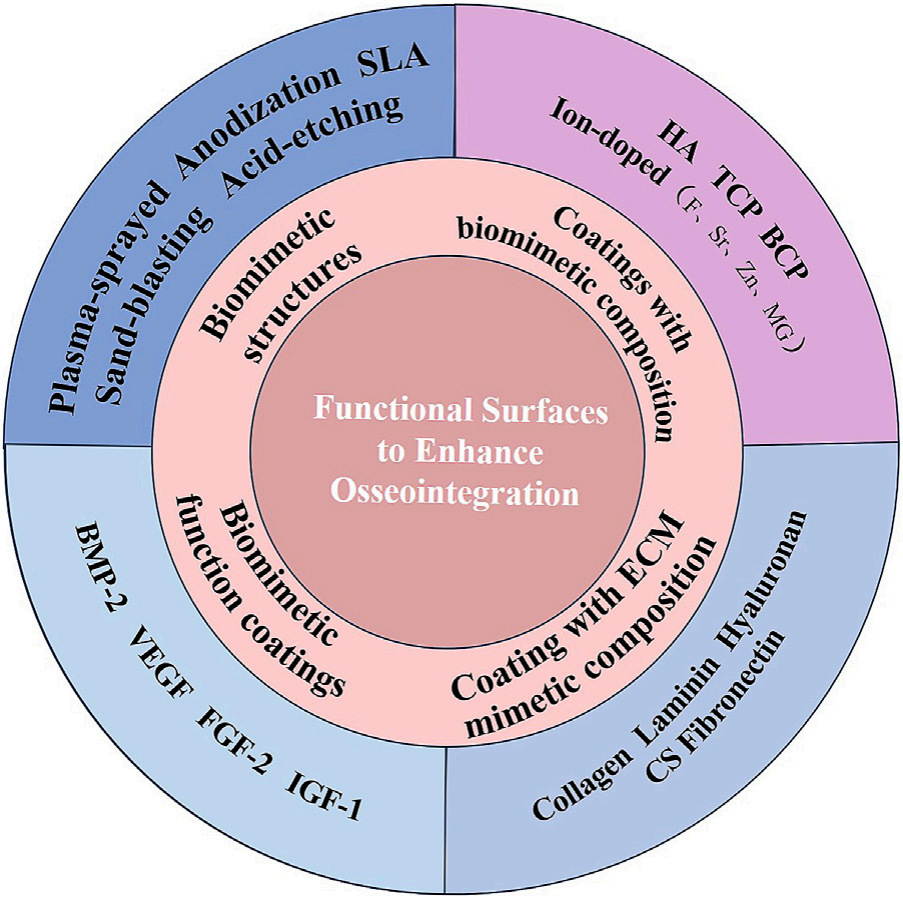
Functional surfaces for dental implants to enhance osseointegration.

To provide new ideas for implant modification with better osseointegration, basic concepts of osseointegration and healing processes after implantation are discussed first. Then, current surface modification methods and emerging biomaterials related to biomimetic structures and biological cues to accelerate osseointegration are reviewed comprehensively (Figure 1).

## 2 Osseointegration and its biological process

Osseointegration is defined as the direct and structural connection between bone and implant without an intermediate layer of connective tissue (Guglielmotti et al., 2019). After being first proposed in the 1960s by Branemark, it has been a revolutionary concept in dental implantology (Brånemark et al., 2001), which is still a hot topic in dental implantology. The osseointegrated dental implants reflect the biological and mechanical fixation of implant fixture into the jaw bone, and the biological fixation is a prerequisite for the long-term success of dental implants.

The process of implant osseointegration is complex and dynamic and takes several weeks of healing. An essential part of osseointegration is the process of bone regeneration, which is regulated by several biological factors (Bosshardt et al., 2017). As reported, there are complex processes underlying bone regeneration, especially the early healing phase and its highly dynamic environment, which impacts the signaling pathways that direct the healing process (Duda et al., 2023). The initial stages of bone healing are characterized by dynamic self-organization of the tissue that governs the healing outcome (Duda et al., 2023). Thus, it is crucial to understand the bone healing process, which may be helpful to understand the critical factors that affect the success of dental implants.

The surface of implants is also a critical factor in affecting the dynamic self-organization of the native bone tissue and their biological responses (Smeets et al., 2016), and thus, it is important to understand the surface properties of dental implants on the results of osseointegration. Since the healing process partially influences bone regeneration and the success of dental implants, the knowledge of the healing process for dental implants may provide us with more endogenous bone regeneration clues and factors to help us to design a better implant surface.

### 2.1 Healing process for dental implants

**FIGURE 2:**
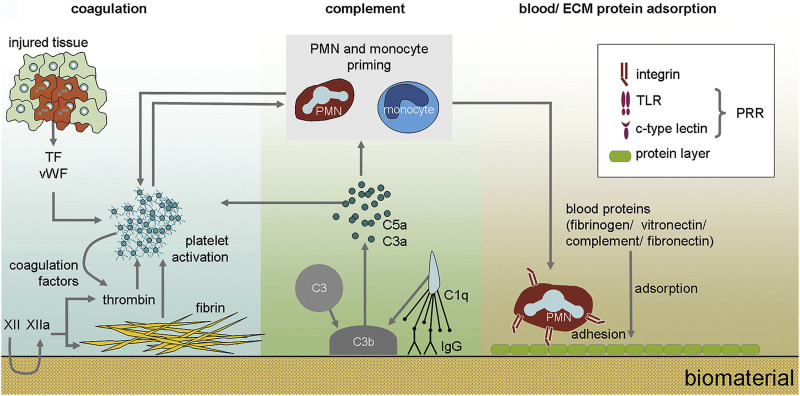
The immediate biological processes after implantation of biomaterials. Nanoseconds after the first contact with tissue, proteins from blood and interstitial fluids adsorb to the biomaterial surface. The adsorption of blood proteins determines the activation of coagulation cascade, complement system, platelets and immune cells and guides their interplay (Franz et al., 2011).

Osseointegration is a time-dependent dynamic process that depends on the biological process of bone healing and the surface properties of dental implants. Generally, when the implant was placed in the bone, several bioresponsive behaviors would take place (Figure 2) (Franz et al., 2011). When the implant is implanted, the contact of implants with blood and tissue fluid will result in the adsorption of proteins on the surface of implants. This layer of proteins determines the activation of the coagulation cascade, complement system, platelets, and immune cells and guides their interplay (Franz et al., 2011). Blood clots form immediately on the surface of dental implants, and platelets are activated to secrete a large number of growth factors for directing preosteoblasts to differentiate into osteoblasts. At this time, the necrotic bone and hematoma are resorbed by osteoclasts and macrophages in granulation tissue, respectively (Chen et al., 2020b). Some special molecules or proteins are adsorbed on the surface of dental implants, which may guide the adhesion of osteoblasts on the surface of dental implants. When osteoblasts are surrounded by their products or matrix, they will be differentiated into osteocytes to promote further bone formation, which means bone is formed on the surface of dental implants. The reaction will continue until the surface of implants is covered by bone (Chen et al., 2020b).

### 2.2 Challenges for dental implants

An ideal dental implant should have great potential to realize osseointegration. The mechanism of osseointegration is closely related to biomaterials designed to be implanted (Guglielmotti et al., 2019). However, the common biomaterials used in dental implants in clinic such as titanium (Ti) and its alloys, are bioinert and has limited biological activity. From the biological point of view, early and late implant loss is considered a failure to achieve or maintain osseointegration, respectively (Tomasi and Derks, 2022). Therefore, enhancing the bioactivity of dental implants is a vital issue that needed to be addressed. Since the healing process is crucial for bone regeneration, we should consider the healing process to improve osseointegration. The factors that enhance the osseointegration may improve the properties of dental implants. Furthermore, we should also consider the factors that can promote bone healing or bone regeneration (Smeets et al., 2016). Therefore, the challenge for dental implants is how to endow biomaterials designed to be implanted with better bioactivity, fully considering the healing process and bone regeneration.

Many review works have been published about the surface modification of dental implants, most of which are focused on the surface modification methods or surface coating materials (Souza et al., 2019; Inchingolo et al., 2023). Herein, we try to consider the bone healing process and connect the biomimetic idea of bone regeneration to discuss the surface modification of dental implants. We aim to review the functional surface modification of dental implants based on the biomimetic concept and provide guidance for optimal osseointegration methods by discussing their biological characteristics.

## 3 Surface modification of dental implants and biomimetics

Generally, the interactions between the implants and bone first happen on the surface of implants. Thus the surface has great effects on the following healing process of implants, such as protein adsorption, activation of coagulation cascade, complement system, platelets, the subsequent immune responses, osteogenesis, and osteointegration (Franz et al., 2011; Smeets et al., 2016; Chen et al., 2020b). Therefore, it is still necessary to tune the surface properties of dental implants to improve its bioproperties and enhance bone regeneration.

The concept of “osseointegration” is related to bone regeneration, a hot topic in recent years. Therefore, the factors that can promote bone regeneration would be critical for osseointegration. It has been critical to use biomaterials to initiate the regeneration of defected bone. The process of osseointegration is closely associated with biomaterials, which are designed for implantation or incorporation into living organisms with the aim of replacing or regenerating tissues and their functions. It is an ideal strategy for designing implant biomaterials with porous structures, biomimetic composition, and biomimetic functions for bone regeneration (Liu et al., 2016). Since surface topography and compositions can significantly affect bone healing and osseointegration, the surface of dental implants is much important. In this part, we will give a summary of the surface modification of dental implants. We mainly consider the problem from the biomimetic idea, which can mimic the natural healing process of dental implants, and the current surface functionalization or coatings to tune the osseointegration will be summarized in the following part.

### 3.1 Biomimetic structures of dental implants

**FIGURE 3:**
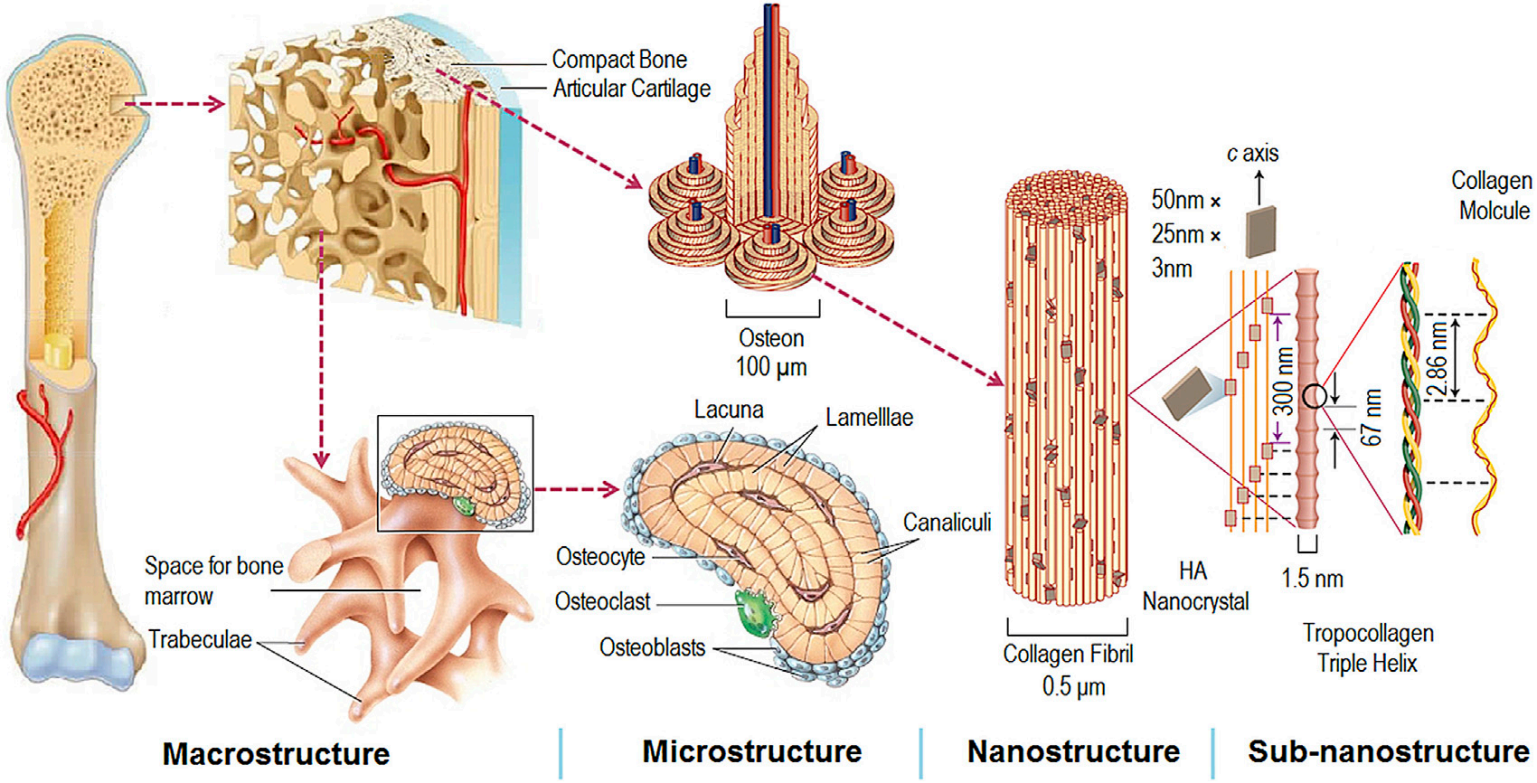
Hierarchical and multiscale structure of natural bone (Wang et al., 2016).

The natural bone consists of micro-nano-scale hierarchical structures, and its main components are hydroxyapatite (HA) and collagen (mostly type I) (Figure 3) (Wang et al., 2016). The biomimetic multiscale structures may supply a preferable microenvironment for bone healing and bone regeneration (Liu et al., 2016). Therefore, many bone tissue engineering scaffolds with porous and micro-nano-scale structure have been designed to improve bone regeneration. It is well known that porous structure, biocompatible scaffolds, and special biofunctions are critical in the field of bone tissue engineering. There are some similarities between the scaffolds and dental implants in achieving ideal bone regeneration. Thus, dental implants with biomimetic porous and micro-nano-scale structures may improve osseointegration and reduce the healing time.

When the implant surface is porous, it may mimic the structure of bone. This biomimetic structure is beneficial for the ingrowth of bone and forms interlocks with the new bone, which may greatly improve osseointegration and thus increase the biomechanical stability and resistance fatigue loading of implants (Hasegawa et al., 2020). Besides, it is widely acknowledged that cellular responses and osseointegration to implants vary depending on the surface roughness at micro-, submicro- and nano-scale levels (Hasegawa et al., 2020). For instance, micro-rough structures favor cell attachment, while nano-rough structures encourage gene expression, protein synthesis, and cell differentiation (Gittens et al., 2011; Gittens et al., 2013). A variety of techniques, including plasma-sprayed (Ferraz et al., 2001; Giavaresi et al., 2003), anodization (Sul et al., 2002; Qi et al., 2021), sand-blasting (Gil et al., 2021; Wang et al., 2023), and acid-etching (Park and Davies, 2000; Trisi et al., 2003), have been employed to create biomimetic porous and rough surfaces of dental implants, and the results have demonstrated that implants with biomimetic structures would have better results for successful implant (López-Valverde et al., 2020).

**FIGURE 4:**
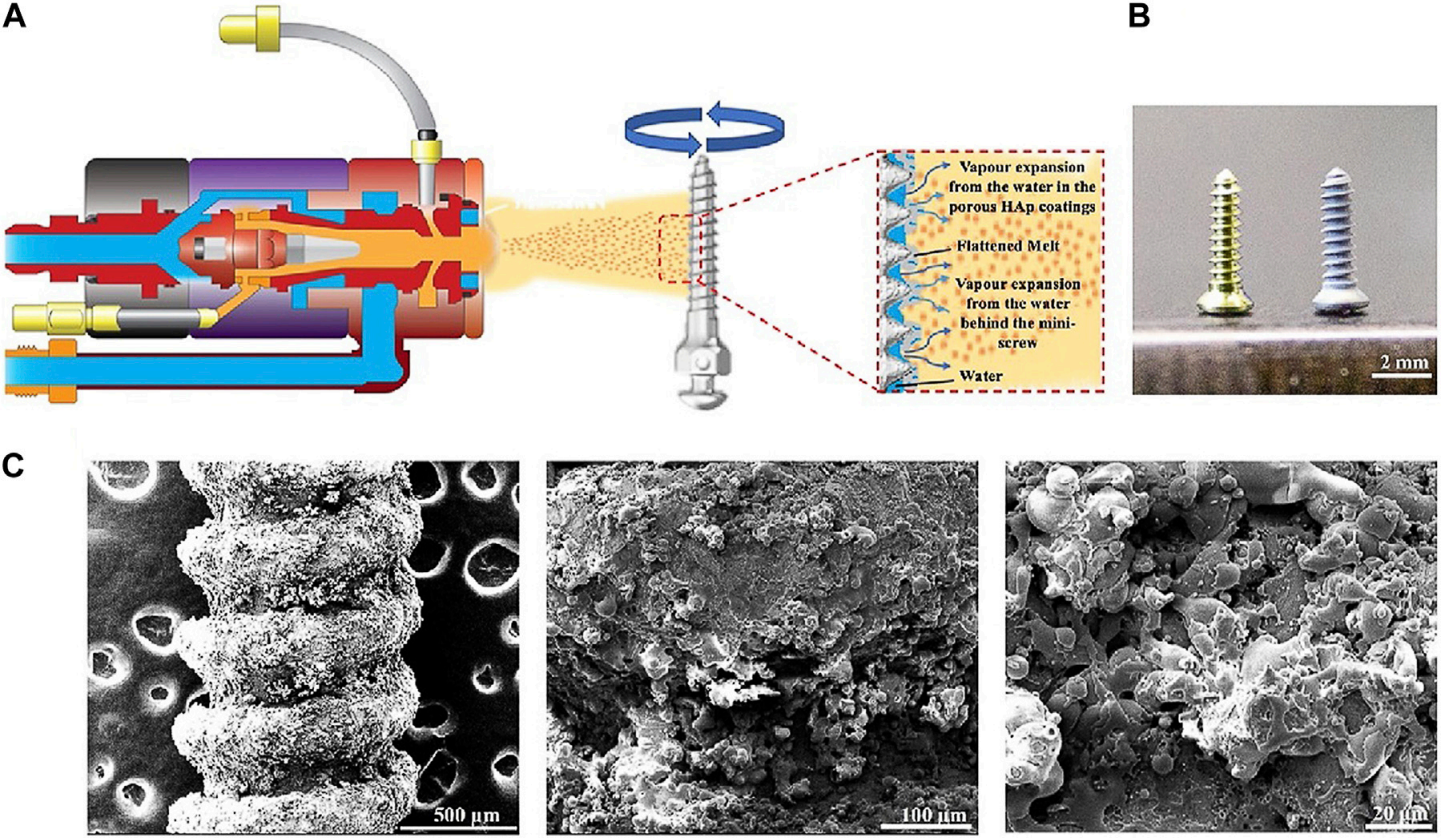
Preparation of HA coatings on Ti implants using the vapor-induced pore-forming atmospheric plasma spraying technique (Hou et al., 2023). **(A)** The Ti implant was roughened and then sprayed with a thin layer of HA coatings. **(B)** Left: Ti implants; right: Ti implants with the porous HA coating. **(C)** SEM of HA coatings on implant surfaces at different magnifications (Hou et al., 2023).

Usually, the plasma-sprayed method is used to coat Ti, HA, and zirconia (ZrO_2_) onto implant surfaces. This method always creates a surface with micro-scale roughness. For example, plasma-sprayed HA coating shows an average surface roughness of about 1.06 μm (Ferraz et al., 2001; Giavaresi et al., 2003), and plasma-sprayed ZrO_2_ coating presents a roughness of about 1.58 µm (Huang et al., 2018b), which are better suitable for osseointegration than uncoated implants. Using the plasma-sprayed method combines with the vapor-induced pore-forming technique, a rough and porous HA coating could be effectively fabricated. The desired thickness of HA coating is achieved by multiple rapid sprays in pure water (Figure 4) (Hou et al., 2023). The resulting Ti coating via the plasma-sprayed method has an average roughness of around 7 μm, which accelerates the bone/implant interface formation (Le Guéhennec et al., 2007). Another common method to prepare micro- or nano-roughness surfaces is the anodization of Ti (Sul et al., 2002; Qi et al., 2021). Anodization leads to modifications in the microstructure and the crystallinity of the titanium oxide layer (Sul et al., 2002), which strengthens the bone response and generates superior results for biomechanical and histomorphometric tests (Le Guéhennec et al., 2007). The nanostructured implant surfaces via anodization present nanotubes of various sizes (Qi et al., 2021). The nanoscale topography of the Ti surface is altered by various anodization voltages, ranging from 30 nm at 5 V to 80 nm at 20 V (Ma et al., 2014). The prepared Ti surfaces’ roughness increased as a result of the increasing anodization voltage. Compared with 80 nm TiO_2_ nanotubular and smooth surfaces, 30 nm TiO_2_ nanotubular coating exhibits lesser pro-inflammatory properties, more bone formation, and better osseointegration (Ma et al., 2014; Qi et al., 2021). Also, 30 nm TiO_2_ surface shows faster collagen synthesis and extracellular matrix (ECM) mineralization in the macrophages’ conditioned media (Ma et al., 2018). After implantation *in vivo*, mineralized bone formation is also significantly faster around the 30 nm TiO_2_ nanotubular surface implant (Ma et al., 2018).

Besides the common plasma-spraying method, the acid-etching and sand-blasting methods are also widely used and often combined to create the appropriate roughness related to the biological response. The acid-etching method usually produces micro-pit structures, with pit sizes ranging from 0.5 to 2 μm (Park and Davies, 2000; Trisi et al., 2003). Park et al. found that acid-etched surfaces enhanced osseointegration by attaching fibrin and osteoblasts around the implant surface (Park and Davies, 2000; Trisi et al., 2003). Sand-blasting method produces a macro-roughness with very sharp peaks and valleys, and many parameters, including the size and nature of abrasive particles, projection pressure, and the distance from the gun to Ti surfaces, affect the roughness of Ti surfaces (Gil et al., 2021). It is widely recognized that acid treatment alone does not create the proper roughness for osseointegration (Gil et al., 2021). Thus, it is often combined with sand-blasting. Herrero-Climent et al. examined the osseointegration of four Ti implants with surfaces that were either as-machined, acid-etched, sand-blasted, or sand-blasted + acid-etched (Herrero-Climent et al., 2013). The sand-blasted with/without acid-etched Ti implants had a higher roughness and better osseointegration than the other two Ti implants. Besides, the findings showed that, in comparison with the sand-blasted implants, the combination of sand-blasted and acid-etched accelerated lightly bone regeneration at various implantation times (Herrero-Climent et al., 2013). Sand-blasted, large-grit, and acid-etched (SLA) technique has emerged as the most commercially successful Ti-based dental implant, which was introduced in 1997. It is a process that involves blasting with coarse abrasive particles followed by acid-etching (Im et al., 2023). SLA generates a topographical surface that exhibits isotropic characteristics. This topography consists of macro-scale irregularities, as well as interconnected cavities at the micro-scale and submicro-scale levels (Kim et al., 2013). The greater osseointegration is believed to be attributed to several factors, including improved mechanical interlocking with the adjacent bone, increased surface area, surface energy, protein adsorption, and cell adhesion during the initial stages of wound healing (Im et al., 2023). Instead of the conventional dual etching solutions consisting of sulphuric and hydrochloric acids used in SLA, Jae-Seung Im et al. used an eco-friendly hydrogen peroxide (H_2_O_2_)/sodium bicarbonate (NaHCO_3_) mixture for etching. Osteoblast adhesion and proliferation were enhanced on the modified SLA surfaces (Im et al., 2023).

While biomimetic structures provide a preferable physical microenvironment, the ability of the sole topography cue to encourage cell attachment and proliferation is constrained in the absence of biological cues like bioactive factors on implants (Li et al., 2021). As a result, the biological modifications on the topological implants have been developed with various osteoinductive biomaterials and bioactive molecules (Agarwal and García, 2015). To date, the composition derived from bone such as HA and collagen, has been immobilized on dental implants for accelerating bone regeneration (Scarano et al., 2019). Besides biomimetic structures, therefore, the surface of dental implants should also mimic the composition of bone or contain osteogenesis-related biofactors to provide sufficient biological cues to promote bone regeneration and osseointegration, and thus biomimetic coatings have been prepared.

### 3.2 Coatings with biomimetic composition

The natural bone is composed of inorganic and organic composition. The typical components are HA, one kind of calcium phosphate (CaP), and collagen, one kind of ECM proteins, respectively. CaP ceramics, known for their superior osteoinductivity, are able to induce ectopic bone formation in non-osseous sites (Chen et al., 2020a; Wang et al., 2022). Besides, ECM is a dynamic structure that is constantly remodelled to regulate tissue homeostasis, and its components represent promising therapeutic targets (Bonnans et al., 2014). Therefore, coating with the biomimetic bone composition, including CaP and ECM components, should be a promising strategy in the field of surface modification of dental implants to obtain superior osseointegration.

#### 3.2.1 Biomimetic inorganic composition

CaP ceramics, the main inorganic composition of bone, are frequently employed as biomimetic coating materials because of their excellent osteoconductivity and osteoinductivity. A carbonate apatite layer that is chemically and crystallographically identical to the inorganic phase of bone forms on implants as a result of an ion exchange reaction between implants and surrounding body fluids. And this carbonate apatite layer aids the bone healing process (de Jonge et al., 2008). Therefore, CaP coatings are widely used to mimic the bone healing process to construct a new bioactive implant surface to facilitate further bone contact. There are a series of CaP ceramics with various Ca/P ratios. For example, monocalcium phosphate anhydrous (Ca(H_2_PO_4_)_2_), dicalcium phosphate anhydrous (CaHPO_4_), tricalcium phosphate (Ca_3_(PO_4_)_2_, TCP), HA (Ca_10_(PO_4_)_6_(OH)_2_), carbonate apatite (Ca_5_(PO_4_)_x_(CO_3_)_y_) present different Ca/P ratio from 0.5 to more than 1.67. These CaP coatings are described to mimic the functions of natural bone and make it easier to bridge small gaps between implants and surrounding bone, thus improving the osseointegration of dental implants (Hayakawa et al., 2000; de Jonge et al., 2008; Tabrizi et al., 2022).

Among various CaP ceramics, HA, or more specifically carbonate apatite, is by far the most abundant inorganic phase of bone. The adhesion of osteoblasts and the mineralization of new bone can be encouraged by the HA coatings. HA coated dental implants can be prepared via kinds of methods, such as plasma-sprayed (Schopper et al., 2005), spin coating (Tredwin et al., 2013), sol-gel dip-coating (Tredwin et al., 2013; Li et al., 2014), electrophoretic deposition (Iwanami-Kadowaki et al., 2021), electrochemical deposition (Zhao et al., 2013), and atomic layer deposition (Kylmäoja et al., 2022). The only industrial process for fabricating HA coatings on orthopedic and dental implants designed for commercialization is the plasma-sprayed technique (Prezas et al., 2023), which makes implants better than uncoated ones (Schopper et al., 2005). However, the plasma-sprayed coating results in phase and structural inhomogeneity and leads to a reduction in cohesion failure at the coating/implant interface (Cheang and Khor, 1996; Tredwin et al., 2013). Besides, a discrepancy in coefficients of thermal expansion between the metal and HA has an impact on the adhesive bond strength of the HA coating (Ke et al., 2019). By using a gradient HA coating created by the laser designed net shaping and plasma spraying, Ke et al. dramatically increased the adhesive bond strength from 26 ± 2 MPa to 39 ± 4 MPa (Ke et al., 2019). On the contrary, due to low temperature processing, the sol-gel dip coating method presents phase and structural homogeneity (Tredwin et al., 2013). Despite being a straightforward and inexpensive method, dip coating has problems when used for complicated shapes, controlling coating thickness, and obtaining enough adhesive strength (Iwanami-Kadowaki et al., 2021). The application of electrophoretic deposition is hampered by the required multiple steps, high temperature, specialized equipment, and demanding conditions. Further sintering at 600°C or higher and voltages of the order of 20–200 V are used to create HA coatings by electrophoretic deposition (Kim and Ramaswamy, 2009). Besides, highly crystalline HA produced at high temperatures is difficult to degrade, exhibits limited biological activity, and has relatively single structures (Zhao et al., 2020). Currently, researchers have been focused on a bioinspired method to synthesize the HA coating to avoid these limitations (Ma et al., 2023).

The common bioinspired method used in HA-coated implants was the polydopamine (PDA)-assisted method (Ma et al., 2023). It is a simple, mild but effective way to prepare HA-coated by the immersion of PDA-modified Ti into simulated body fluid (SBF). In this process, PDA provides numerous nucleation sites for mineralization and spontaneously reacts with Ca and P ions in SBF, thus leading to HA deposition (Zhe et al., 2016). While HA formed at high temperatures is high crystalline, HA prepared by the PDA-assisted bioinspired method has spherical particle structures and better bioactivity (Xu et al., 2018). Contrary to the aforementioned methods, which frequently resulted in cohesion failure at the coating/implant interface, the PDA-assisted bioinspired method leads to a stable HA coating on implants owing to the superior adhesion properties of PDA (Xu et al., 2018). It is reported that HA coatings remain stable even after strong ultrasonication for 1 h (Xu et al., 2018).

There are many other ions contained in the composition of natural bone, and to mimic the natural composition, various ions such as fluoride (F), strontium (Sr), zinc (Zn), magnesium (Mg) ions have been doped into HA crystals and formed ions doped HA, such as F-HA and Sr-HA have been designed to work as coatings (Bonnelye et al., 2008; Tredwin et al., 2013; Li et al., 2014; Panzavolta et al., 2018). These doped ions are also necessary in the norm physiological system, and thus it is well to design coatings that mimic the composition in the body. The ions can work as they are and show great potential in bone regeneration and thus promote osseointegration. Many studies show that ion-doped HA increased biological efficiency instead of pure HA (Bonnelye et al., 2008; Li et al., 2014). For example, Tredwen et al. compared the potential bond strength and interaction of HA, F-doped HA, and fluarapatite (FA) with Ti, and it was found that increasing F^−^ substitution significantly increased bond strength (Tredwin et al., 2013). Sr-doped HA coating significantly promotes the proliferation and differentiation of bone mesenchymal stem cells (BMSCs) and osteoblasts when compared with untreated and HA-coated Ti surfaces (Panzavolta et al., 2018). Due to the different radii and properties of the two atoms, the lattice of Sr-doped HA can be distorted, and the biodegradability increases (Li et al., 2007). In addition to the better osseointegration with improved trabecular parameters and higher bone-to-implant contact (BIC) (Zhao et al., 2013), Mg-doped HA coating increases the maximum push-out force and interfacial shear strength compared to HA coatings (Li et al., 2014). To combine the different benefits of various ions, Hou et al. prepared Zn-, Sr- and Mg-multidoped HA (ZnSrMg-HA) porous coatings on implants. ZnSrMg-HA coating showed the most pronounced osteogenesis and concentrated bone growth along implant treads when compared with HA and Zn-doped HA groups (Hou et al., 2023).

Similar to HA, other CaP ceramics such as TCP and biphasic calcium phosphate (BCP) also show superior osteoinductivity and osteoconductivity, and thus, they are widely used as coating materials to modify the surface of dental implants to improve osseointegration (Hayakawa et al., 2000; Tabrizi et al., 2022). It is found that CaP sputter coated implants always show a higher BIC than the non-coated implants *in vivo* (Hayakawa et al., 2000). Besides, BCP coating makes the secondary stability of implants much higher (Tabrizi et al., 2022). Despite the resorption rate of TCP higher than HA, it is still often lower than the rate of new bone formation (Damerau et al., 2022). The ionic substitutions such as silver (Ag), Zn, or copper (Cu) can increase the resorption rate and provide other functional properties to TCP, including antibacterial activity (Fadeeva et al., 2021). From a systematic review in a meta-analysis, there does not seem to be much effect of TCP-coated implants over uncoated implants in the short term, however, there was an increase in differences in BIC for TCP-coated implants over time (Damerau et al., 2022).

In general, the application of biomimetic CaP coatings, especially HA, on Ti implants has proved their effectiveness in promoting osseointegration. The chemical structure, composition, ion doping, and other characteristics of the manufactured CaP coatings vary greatly. Despite the proven efficacy of CaP coatings, the method to fabricate CaP coatings is still a key step for success of dental implants.

#### 3.2.2 ECM biomimetic coatings

During the healing process, osteoblasts adhere to the surface of dental implants, and then proliferation and differentiation happens, which is followed by a series of regeneration process. It is critical to prepare dental implants with a special environment that may be suitable for the growth of osteoblasts and other osteogenic cells. As reported, the behavior of stromal cells and bone healing are affected by the newly forming ECM (Duda et al., 2023). Through ECM, the cell-generated forces are directly transmitted to neighboring cells, and this transmission is highly influenced by ECM composition, which is important in determining the success of bone healing (Duda et al., 2023). That is, ECM has pronounced impacts on guiding bone healing by providing an environment for cellular responses. Thus, a promising method to enhance osseointegration is to coat dental implants with ECM components (Schulz et al., 2014).

Collagen type I (COL1), the main organic composition of natural bone and ECM, is a good choice to act as functional coatings on the surface of dental implants to mimic the natural interface, which promote the adhesion of osteoblasts, and finally improve bone mineralization and osseointegration (Scarano et al., 2019). After coating with COL1, implants present more trabeculae bone in the implant concavities with a small medullary space when compared with the uncoated group (Scarano et al., 2019). The bioactivity, BIC, and bone areas around the implant surfaces are significantly improved, which could be clinically advantageous for shortening the implant healing period (Scarano et al., 2019). COL1 coatings interact with the integrin receptors, and activate the FAK/PI3K/MAPK pathway of BMSCs, thus leading to promotion of cell proliferation and mineralization of the ECM (Hsu et al., 2019). Importantly, Ti implants coated with COL1 are effective in promoting implant osseointegration *in vivo* (Cho et al., 2021), even in compromised bone such as in the osteopenic rat animal models (Sartori et al., 2015). In addition to promoting osteogenesis, COL1 coating can support macrophage timely conversion from the pro-inflammatory to the pro-healing phenotype, and foster a favorable osteoimmune microenvironment (Shao et al., 2022; Zhao et al., 2022). Since HA and COL1 are the most important components of bone, the construction of HA/COL1 coating is a good alternative to mimic the natural bone. Many studies show that HA/COLl or mineralized collagen coating significantly improves the nucleation ability in SBF and bioactivity of implants (Patty et al., 2022), and leads to more rapid osseointegration (Iwanami-Kadowaki et al., 2021).

Generally, two kinds of methods are used to prepare collagen-coated implants, including physical adsorption and chemical covalent bonding methods. The former relies on van der Walls forces, hydrophilicity, and electrostatic forces (Ao et al., 2014). One of the limitations of this technique is that it is unable to handle the fixing and releasing processes of biomolecules on the implant surfaces due to their weak interactions (Lupi et al., 2021). Thus, the originally adsorbed biomolecules could be quickly desorbed from the surface (Lupi et al., 2021; Zhao et al., 2022). The chemical covalent bonding method involves the use of cross-linking agents, such as aminopropylsilane, glutaraldehyde (GA), or carbodiimide (Ao et al., 2014). Besides, gamma-rays (GRs) could also be exploited to cross-link collagen on Ti surfaces, which leads to a similar performance in new bone areas and BIC with GA crosslinked COL1 coating (Cho et al., 2021). The covalently immobilized Ti coating had more collagen than the physically absorptive one, which increases its ability to regulate BMSCs’ osteogenic activity (Ao et al., 2014). Silanization is a common way to chemically immobilize collagen onto implants (Lupi et al., 2021), which always changes the distribution and conformation of COL1 on surfaces (Marín-Pareja et al., 2015). After silanization treatment, COL1-coated Ti organizes in globular clusters rather than fibrilllar networks. It results in improved fibroblast adhesion, better cell spreading, and stronger fibronectin fibrillogenesis (Marín-Pareja et al., 2015). In addition to silanization, procyanidin is also employed as a natural cross-linker to immobilize COL1 on implant surfaces (Hsu et al., 2019). Procyanidin, a polyphenolic molecule from natural sources like grapeseed, is less toxic than the widely used cross-linker GA (Han et al., 2003). Previous studies have shown that the abundant hydroxyl groups in procyanidin form hydrogen bonds with COL1 to achieve cross-linking without destroying the collagen structure (He et al., 2011). Besides, genipin obtained from the fruit of Genipa Americana may be an alternative of natural crosslinking agents (Liu et al., 2021). It is worth noting that the PDA-assisted bioinspired method can also be used to covalently immobilize with COL1 on Ti surfaces (Zhao et al., 2022).

**FIGURE 5:**
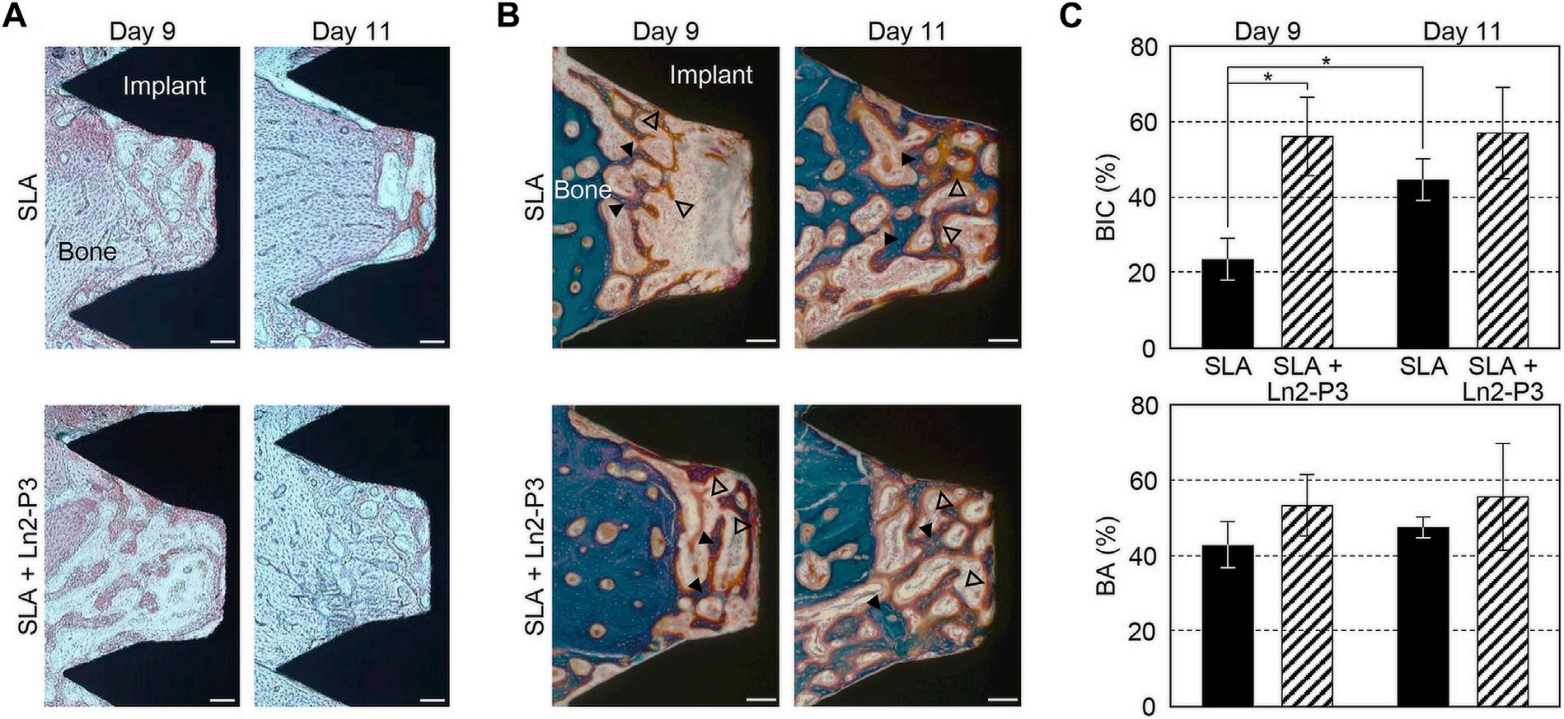
Alizarin red staining Images **(A)**, Masson trichrome-stained images **(B)**, BIC (top) and bone area (bottom) ratios **(C)** of the untreated (SLA) and Ln2-P3-treated (SLA + Ln2-P3) implants in the rabbit tibia at 9 and 11 days after insertion (Choi et al., 2020).

Besides, there were many other important organic components of ECM. For example, laminin, hyaluronan, chondroitin sulfate (CS) and fibronectin, are all biocompatible materials and have been used to improve the surface properties of dental implants (Bougas et al., 2012; Yeo et al., 2015; Chang et al., 2016; Aung et al., 2023). It is reported that laminin coatings induce faster osseointegration around Ti implants both *in vitro* and *in vivo* (Bougas et al., 2012; Yeo et al., 2015). Jung-Yoo Choi et al. evaluated the impact of the Ln2-P3 peptide, generated from laminin, on the osseointegration of implants in rabbit models. The BIC and bone area of the Ln2-P3-coated implants were found to be considerably higher when compared with the uncoated implants on Day 9 after implantation (Figure 5) (Choi et al., 2020). Although several studies indicated the potential interest of hyaluronan coating on Ti surfaces, a crossover randomized clinical trial up to 36 months after loading showed that there were no differences in the healing and implant success between the hyaluronan coated and control implants (Lupi et al., 2019).

Rather than a single ECM component coated on implant surfaces, several components used together may exert better osseointegration. For example, higher BIC was observed in the COL1/low sulfated hyaluronan coated Ti implants when compared with commercial pure Ti implants in the early healing period (Schulz et al., 2014). Haiyong Ao et al. developed a novel stable collagen/hyaluronan multilayer covalent-immobilized coating on Ti implants by the combination of LBL and covalent immobilization technique. When compared with collagen/hyaluronan multilayer absorbed Ti coatings, the multilayer covalent-immobilized coating showed favorable stability and better osteogenesis performance both *in vitro* and *in vivo* (Ao et al., 2013; Ao et al., 2018). It has been demonstrated that collagen/CS coatings affect osteoblast adhesion and BMSCs differentiation (Stadlinger et al., 2012). Interestingly, an increased concentration of CS was unable to enhance this impact for the fact that more CS would desorb from the collagen coating correspondingly (Stadlinger et al., 2012). To find out if the collagen-CS coating affected osseointegration, Kellesarian et al. conducted a systematic review and meta-analysis. The collagen-CS coated implants were reported to have superior new bone formation and BIC, and/or bone volume density (Kellesarian et al., 2018). According to the experimental data, osseointegration appeared to be aided by the collagen-CS coating (Kellesarian et al., 2018). Also, the combination of collagen type II and CS could exhibit a positive influence on bone formation after coating on the implant surface (de Barros et al., 2015).

In summary, ECM component coatings exhibit favorable osseointegration, especially when several components were used together. It is worth noting that chemical covalent bonding methods are preferable to physical adsorption methods. ECM biomimetic coating exerts its impact on osseointegration by mimicking the natural interface and providing an environment to influence the response of osteoblasts, thereby its effectiveness is weaker than that of biofunction coatings which directly induce or determine bone regeneration.

#### 3.2.3 Biomimetic function coatings

Although the biomimetic structures or ECM components can promote osseointegration, it is still difficult to mimic the special biofunctions related to the healing process. Since the cell functions and growth factors are essential to bone regeneration, many methods have been employed to prepare the growth factor coated dental implants to mimic the biofunctions and promote bone regeneration. The growth factors can control osteogenesis, ECM formation, and bone regeneration by affecting the recruitment and differentiation of osteoprogenitor cells (Bose et al., 2012). From the point of bone regeneration, although ECM has great potential, the growth factors such as bone morphogenic protein-2 (BMP-2) (Liu et al., 2007; Kim et al., 2015), vascular endothelial growth factor (VEGF) (Leedy et al., 2014; Schliephake et al., 2015), fibroblast growth factor-2 (FGF-2) (Nagayasu-Tanaka et al., 2017; Yasunaga et al., 2022), insulin-like growth factor-1 (IGF-1) (Raju et al., 2023) are also much important in bone tissue regeneration. These growth factors have been recognized as critical factors. Since dental implants are also bone regeneration related, it is a good method to coat the dental implants with these bioactive factors to mimic their special biofunctions.

**FIGURE 6:**
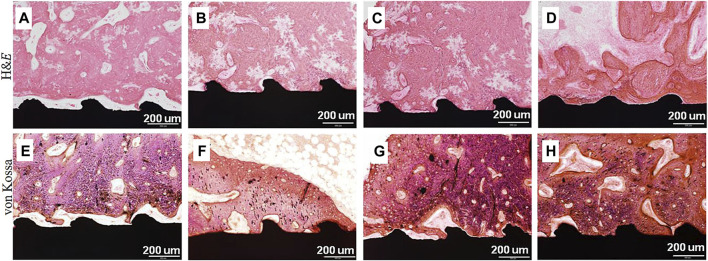
H&E and von Kossa stained images on the surrounding tissue around **(A,E)** pristine Ti, **(B,F)** Ti/HA, **(C,G)** Ti/Hep/BMP-2, and **(D,H)** Ti/HA/Hep/BMP-2 samples 4 weeks after implantation (Yang et al., 2015).

BMP-2 is a known and effective osteogenic agent and has been approved by the Food and Drug Administration (FDA). Its biological effects are dosage-dependent (Kim et al., 2015). Six types of Ti implants, including uncoated, CaP-coated, BMP-2 adsorbed to uncoated, BMP-2 adsorbed to CaP-coated, BMP-2 incorporated into CaP-coated, and BMP-2 adsorbed to and incorporated into CaP-coated, were implanted in the maxillae of minipigs. After 3 weeks, the groups with no BMP-2 presented the most bone volume and bone coverage. Conversely, implants containing only adsorbed BMP-2 exhibited the lowest bone coverage (Liu et al., 2007). It suggested that osteoconductivity of implants was most severely impaired when BMP-2 was only superficially adsorbed on surfaces, and least so when it was incorporated into a CaP coating (Liu et al., 2007). Hunziker’s study also demonstrated that the mode of delivery greatly affected the capacity of BMP-2 to induce and sustain local bone formation. Compared with the adsorbed way, the incorporated way always led to a gradual release and a superior osteogenic response (Hunziker et al., 2012). Rather than direct adsorption of BMP-2, George Calin Dindelegan et al. developed a novel complex coating on Ti implants consisting of a chitosan film engulfing microsphere loaded with BMP-2 (Dindelegan et al., 2021), which could effectively release BMP-2 in a stable and active form that assured short and effective osseointegration (Dindelegan et al., 2021). Chien et al. developed the RGD/HA/BMP-2 coating on Ti implants via the PDA-assisted bioinspired method, and the results indicated that the conjugation of RGD enhanced the adhesion of BMSCs, while the incorporation of HA facilitated cellular osteodifferentiation (Chien and Tsai, 2013), and the immobilization of BMP-2 stimulated osteogenesis of the stem cells. The functionalized coating improved osteogenic differentiation and mineralization (Chien and Tsai, 2013). Yang et al. compared surface-modified Ti samples with HA and heparin (Hep)-BMP-2 complex (Ti/HAp/Hep/BMP-2), Ti/HAp/Hep/BMP-2 samples produced the largest scale of osteons and the maximum number of osteocytes at the interface (Figure 6) (Yang et al., 2015).

VEGF coating on Ti implants has also drawn much attention to improve osseointegration, since vascularization is a crucial prerequisite for bone healing and osseointegration (Leedy et al., 2014; Schliephake et al., 2015). In comparison to uncoated implants, Leedy et al. prepared the VEGF-loaded chitosan coatings on Ti, and found a 2-fold increase in ALP activity and a 10-fold increase in calcium deposition (Leedy et al., 2014). However, around 75% of VEGF was released over the first 12 h, and by day 3, the coatings had released 90%–95% of VEGF. There was a need to reduce the burst release of VEGF and enhance the elution profile (Leedy et al., 2014). DNA oligonucleotide (ODN) strands nano-anchored to Ti surfaces can be used to bind VEGF that has been conjugated to oligonucleotides (Schliephake et al., 2012). After the covalent binding of VEGF on the surface of implants, BIC after 1 month was considerably higher compared to uncoated and ODN strands anchored implants (Schliephake et al., 2015).

Combining BMP-2 and VEGF has been shown to improve bone regeneration and vascularization when compared to using either BMP-2 or VEGF alone (Ramazanoglu et al., 2011). Ramazanoglu et al. studied whether coating with BMP-2 and VEGF affected osseointegration in pigs (Ramazanoglu et al., 2011). There was a notable enhancement in the BV density in the BMP-2 and BMP-2 + VEGF groups at 2 weeks. In contrast, the group treated with BMP + VEGF did not exhibit a statistically significant improvement in BIC at 4 weeks. These suggested that the biomimetic CaP coated implant surfaces along with the addition of BMP and VEGF resulted in increased BV density but not BIC (Ramazanoglu et al., 2011). Interestingly, in the rabbit models after receiving radiotherapy, the combined delivery of BMP-2 and VEGF enhanced bone formation around implants, promoted BIC, and enhanced the stability of implants in irradiated bone (Huang et al., 2018a).

Similar to the coating methods of biomimetic ECM components, covalent binding is better than physical adsorption for fabricating the growth factors functionalized coatings. The burst release of the adsorbed growth factors even impairs the osteoconductivity of implants. Growth factors immobilized on implants pre-coated with CaP are preferable for their effectiveness in inducing bone formation and osseointegration.

## 4 Conclusion and perspective

Herein, we have summarized the biomimetic approaches to improve osseointegration including the structures and various biomimetic coatings. Regarding the bone healing process, micro-nano-scale and porous structures were preferable. Thus, we have summarized the common techniques to achieve an appropriate structure. Besides, how to mimic the composition of bone and construct the biomimetic function coating were also concluded herein. Also, these modification methods and emerging biomaterials’ effects on osseointegration were discussed. The evolution of dental implants has been largely influenced by the integration of new materials and technologies. However, it is still challenging to fabricate uniform biomimetic structures rapidly and on a large scale. With the development of additive manufacturing or three-dimensional (3D) printing technologies, which could provide customized implants, the future of implants should aim to develop surfaces with controlled, refined, and standardized roughness and morphology. Although many traditional or emerging methods were reported very useful on experimental grounds, there were sometimes no significant differences in a systematic review and meta-analysis. Therefore, how to build a multifunctional modified implant surface to meet clinical needs is still on demand. In addition, the immune microenvironment at the implant-bone interface should be added in the future considering.

## References

[B1] Agarwal R. García A. J. (2015). Biomaterial strategies for engineering implants for enhanced osseointegration and bone repair. Adv. Drug Deliv. Rev. 94, 53–62. 10.1016/j.addr.2015.03.013 25861724 PMC4598264

[B2] Alghamdi H. S. Jansen J. A. (2020). The development and future of dental implants. Dent. Mater J. 39, 167–172. 10.4012/dmj.2019-140 31969548

[B3] Ao H. Xie Y. Tan H. Wu X. Liu G. Qin A. (2014). Improved hMSC functions on titanium coatings by type I collagen immobilization. J. Biomed. Mater Res. A 102, 204–214. 10.1002/jbm.a.34682 23661627

[B4] Ao H. Xie Y. Tan H. Yang S. Li K. Wu X. (2013). Fabrication and *in vitro* evaluation of stable collagen/hyaluronic acid biomimetic multilayer on titanium coatings. J. R. Soc. Interface 10, 20130070. 10.1098/rsif.2013.0070 23635490 PMC3673146

[B5] Ao H. Zong J. Nie Y. Wan Y. Zheng X. (2018). An *in vivo* study on the effect of coating stability on osteointegration performance of collagen/hyaluronic acid multilayer modified titanium implants. Bioact. Mater 3, 97–101. 10.1016/j.bioactmat.2017.07.004 29744446 PMC5935658

[B6] Aung L. M. Lin J. C. Salamanca E. Wu Y. F. Pan Y. H. Teng N. C. (2023). Functionalization of zirconia ceramic with fibronectin proteins enhanced bioactivity and osteogenic response of osteoblast-like cells. Front. Bioeng. Biotechnol. 11, 1159639. 10.3389/fbioe.2023.1159639 37180046 PMC10167021

[B7] Bonnans C. Chou J. Werb Z. (2014). Remodelling the extracellular matrix in development and disease. Nat. Rev. Mol. Cell Biol. 15, 786–801. 10.1038/nrm3904 25415508 PMC4316204

[B8] Bonnelye E. Chabadel A. Saltel F. Jurdic P. (2008). Dual effect of strontium ranelate: stimulation of osteoblast differentiation and inhibition of osteoclast formation and resorption *in vitro* . Bone 42, 129–138. 10.1016/j.bone.2007.08.043 17945546

[B9] Bose S. Roy M. Bandyopadhyay A. (2012). Recent advances in bone tissue engineering scaffolds. Trends Biotechnol. 30, 546–554. 10.1016/j.tibtech.2012.07.005 22939815 PMC3448860

[B10] Bosshardt D. D. Chappuis V. Buser D. (2017). Osseointegration of titanium, titanium alloy and zirconia dental implants: current knowledge and open questions. Periodontol 73, 22–40. 10.1111/prd.12179 28000277

[B11] Bougas K. Stenport V. F. Currie F. Wennerberg A. (2012). Laminin coating promotes calcium phosphate precipitation on titanium discs *in vitro* . J. Oral Maxillofac. Res. 2, e5. 10.5037/jomr.2011.2405 24422002 PMC3886082

[B12] Brånemark R. Brånemark P. I. Rydevik B. Myers R. R. (2001). Osseointegration in skeletal reconstruction and rehabilitation: a review. J. Rehabil. Res. Dev. 38, 175–181. 11392650

[B13] Buser D. Sennerby L. De Bruyn H. (2017). Modern implant dentistry based on osseointegration: 50 years of progress, current trends and open questions. Periodontol. 2000 73, 7–21. 10.1111/prd.12185 28000280

[B14] Chang Y. C. Ho K. N. Feng S. W. Huang H. M. Chang C. H. Lin C. T. (2016). Fibronectin-grafted titanium dental implants: an *in vivo* study. Biomed. Res. Int. 2016, 1–11. 10.1155/2016/2414809 PMC4913050 27366739

[B15] Cheang P. Khor K. A. (1996). Addressing processing problems associated with plasma spraying of hydroxyapatite coatings. Biomaterials 17, 537–544. 10.1016/0142-9612(96)82729-3 8991486

[B16] Chen X. Wang M. Chen F. Wang J. Li X. Liang J. (2020a). Correlations between macrophage polarization and osteoinduction of porous calcium phosphate ceramics. Acta Biomater. 103, 318–332. 10.1016/j.actbio.2019.12.019 31857257

[B17] Chen Y. F. Goodheart C. Rua D. (2020b). The body's cellular and molecular response to protein-coated medical device implants: a review focused on fibronectin and BMP proteins. Int. J. Mol. Sci. 21, 8853. 10.3390/ijms21228853 33238458 PMC7700595

[B18] Chien C. Y. Tsai W. B. (2013). Poly(dopamine)-assisted immobilization of Arg-Gly-Asp peptides, hydroxyapatite, and bone morphogenic protein-2 on titanium to improve the osteogenesis of bone marrow stem cells. ACS Appl. Mater Interfaces 5, 6975–6983. 10.1021/am401071f 23848958

[B19] Cho W. T. Kim S. Y. Jung S. I. Kang S. S. Kim S. E. Hwang S. H. (2021). Effects of gamma radiation-induced crosslinking of collagen type I coated dental titanium implants on osseointegration and bone regeneration. Mater. (Basel) 14, 3268. 10.3390/ma14123268 PMC8231814 34199187

[B20] Choi J. Y. Kim S. Jo S. B. Kang H. K. Jung S. Y. Kim S. W. (2020). A laminin-211-derived bioactive peptide promotes the osseointegration of a sandblasted, large-grit, acid-etched titanium implant. J. Biomed. Mater Res. A 108, 1214–1222. 10.1002/jbm.a.36895 32034938

[B21] Damerau J. M. Bierbaum S. Wiedemeier D. Korn P. Smeets R. Jenny G. (2022). A systematic review on the effect of inorganic surface coatings in large animal models and meta-analysis on tricalcium phosphate and hydroxyapatite on periimplant bone formation. J. Biomed. Mater Res. B Appl. Biomater. 110, 157–175. 10.1002/jbm.b.34899 34272804 PMC9292919

[B22] De Barros R. R. Novaes A. B. Jr. Korn P. Queiroz A. De Almeida A. L. Hintze V. (2015). Bone Formation in a local defect around dental implants coated with extracellular matrix components. Clin. Implant Dent. Relat. Res. 17, 742–757. 10.1111/cid.12179 24283497

[B23] De Jonge L. T. Leeuwenburgh S. C. Wolke J. G. Jansen J. A. (2008). Organic-inorganic surface modifications for titanium implant surfaces. Pharm. Res. 25, 2357–2369. 10.1007/s11095-008-9617-0 18509601

[B24] Dindelegan G. C. Caziuc A. Brie I. Soritau O. Dindelegan M. G. Bintintan V. (2021). Multilayered porous titanium-based 3rd generation biomaterial designed for endosseous implants. Mater. (Basel) 14, 1727. 10.3390/ma14071727 PMC8036277 33807480

[B25] Duda G. N. Geissler S. Checa S. Tsitsilonis S. Petersen A. Schmidt-Bleek K. (2023). The decisive early phase of bone regeneration. Nat. Rev. Rheumatol. 19, 78–95. 10.1038/s41584-022-00887-0 36624263

[B26] Fadeeva I. V. Lazoryak B. I. Davidova G. A. Murzakhanov F. F. Gabbasov B. F. Petrakova N. V. (2021). Antibacterial and cell-friendly copper-substituted tricalcium phosphate ceramics for biomedical implant applications. Mater. Sci. Eng. C 129, 112410. 10.1016/j.msec.2021.112410 34579919

[B27] Ferraz M. P. Monteiro F. J. Serro A. P. Saramago B. Gibson I. R. Santos J. D. (2001). Effect of chemical composition on hydrophobicity and zeta potential of plasma sprayed HA/CaO-P2O5 glass coatings. Biomaterials 22, 3105–3112. 10.1016/s0142-9612(01)00059-x 11603581

[B28] Fischer K. Stenberg T. (2012). Prospective 10-year cohort study based on a randomized controlled trial (RCT) on implant-supported full-arch maxillary prostheses. Part 1: sandblasted and acid-etched implants and mucosal tissue. Clin. Implant Dent. Relat. Res. 14, 808–815. 10.1111/j.1708-8208.2011.00389.x 22008715

[B29] Franz S. Rammelt S. Scharnweber D. Simon J. C. (2011). Immune responses to implants - a review of the implications for the design of immunomodulatory biomaterials. Biomaterials 32, 6692–6709. 10.1016/j.biomaterials.2011.05.078 21715002

[B30] Giavaresi G. Fini M. Cigada A. Chiesa R. Rondelli G. Rimondini L. (2003). Mechanical and histomorphometric evaluations of titanium implants with different surface treatments inserted in sheep cortical bone. Biomaterials 24, 1583–1594. 10.1016/s0142-9612(02)00548-3 12559818

[B31] Gil J. Pérez R. Herrero-Climent M. Rizo-Gorrita M. Torres-Lagares D. Gutierrez J. L. (2021). Benefits of residual aluminum oxide for sand blasting titanium dental implants: osseointegration and bactericidal effects. Mater. (Basel) 15, 178. 10.3390/ma15010178 PMC8746027 35009326

[B32] Gittens R. A. Mclachlan T. Olivares-Navarrete R. Cai Y. Berner S. Tannenbaum R. (2011). The effects of combined micron-/submicron-scale surface roughness and nanoscale features on cell proliferation and differentiation. Biomaterials 32, 3395–3403. 10.1016/j.biomaterials.2011.01.029 21310480 PMC3350795

[B33] Gittens R. A. Olivares-Navarrete R. Cheng A. Anderson D. M. Mclachlan T. Stephan I. (2013). The roles of titanium surface micro/nanotopography and wettability on the differential response of human osteoblast lineage cells. Acta Biomater. 9, 6268–6277. 10.1016/j.actbio.2012.12.002 23232211 PMC3618468

[B34] Guglielmotti M. B. Olmedo D. G. Cabrini R. L. (2019). Research on implants and osseointegration. Periodontol. 2000 79, 178–189. 10.1111/prd.12254 30892769

[B35] Gulati K. Chopra D. Kocak-Oztug N. A. Verron E. (2023). Fit and forget: the future of dental implant therapy via nanotechnology. Adv. Drug Deliv. Rev. 199, 114900. 10.1016/j.addr.2023.114900 37263543

[B36] Han B. Jaurequi J. Tang B. W. Nimni M. E. (2003). Proanthocyanidin: a natural crosslinking reagent for stabilizing collagen matrices. J. Biomed. Mater Res. A 65, 118–124. 10.1002/jbm.a.10460 12635161

[B37] Hasegawa M. Saruta J. Hirota M. Taniyama T. Sugita Y. Kubo K. (2020). A newly created meso-micro-and nano-scale rough titanium surface promotes bone-implant integration. Int. J. Mol. Sci. 21, 783. 10.3390/ijms21030783 31991761 PMC7036846

[B38] Hayakawa T. Yoshinari M. Nemoto K. Wolke J. G. Jansen J. A. (2000). Effect of surface roughness and calcium phosphate coating on the implant/bone response. Clin. Oral Implants Res. 11, 296–304. 10.1034/j.1600-0501.2000.011004296.x 11168222

[B39] He L. Mu C. Shi J. Zhang Q. Shi B. Lin W. (2011). Modification of collagen with a natural cross-linker, procyanidin. Int. J. Biol. Macromol. 48, 354–359. 10.1016/j.ijbiomac.2010.12.012 21185325

[B40] Herrero-Climent M. Lázaro P. Vicente Rios J. Lluch S. Marqués M. Guillem-Martí J. (2013). Influence of acid-etching after grit-blasted on osseointegration of titanium dental implants: *in vitro* and *in vivo* studies. J. Mater Sci. Mater Med. 24, 2047–2055. 10.1007/s10856-013-4935-0 23625320

[B41] Hou H. H. Lee B. S. Liu Y. C. Wang Y. P. Kuo W. T. Chen I. H. (2023). Vapor-induced pore-forming atmospheric-plasma-sprayed zinc-strontium-and magnesium-doped hydroxyapatite coatings on titanium implants enhance new bone formation-an *in vivo* and *in vitro* investigation. Int. J. Mol. Sci. 24, 4933. 10.3390/ijms24054933 36902368 PMC10003357

[B42] Hsu C. M. Sun Y. S. Huang H. H. (2019). Enhanced cell response to zirconia surface immobilized with type I collagen. J. Dent. Res. 98, 556–563. 10.1177/0022034519828702 30786812

[B43] Huang B. Yao Q. Huang Y. Zhang L. Yao Y. Gong P. (2018a). Combination use of BMP2 and VEGF165 promotes osseointegration and stability of titanium implants in irradiated bone. Biomed. Res. Int. 2018, 1–11. 10.1155/2018/8139424 PMC6304532 30627574

[B44] Huang Z. Wang Z. Li C. Yin K. Hao D. Lan J. (2018b). Application of plasma-sprayed zirconia coating in dental implants: study in implants. J. Oral Implantol. 44, 102–109. 10.1563/aaid-joi-d-17-00020 29303416

[B45] Hunziker E. B. Enggist L. Küffer A. Buser D. Liu Y. (2012). Osseointegration: the slow delivery of BMP-2 enhances osteoinductivity. Bone 51, 98–106. 10.1016/j.bone.2012.04.004 22534475

[B46] Im J. S. Choi H. An H. W. Kwon T. Y. Hong M. H. (2023). Effects of surface treatment method forming new nano/micro hierarchical structures on attachment and proliferation of osteoblast-like cells. Mater. (Basel) 16, 5717. 10.3390/ma16165717 PMC10456429 37630008

[B47] Inchingolo A. M. Malcangi G. Ferrante L. Del Vecchio G. Viapiano F. Inchingolo A. D. (2023). Surface coatings of dental implants: a review. J. Funct. Biomater. 14, 287. 10.3390/jfb14050287 37233397 PMC10218820

[B48] Iwanami-Kadowaki K. Uchikoshi T. Uezono M. Kikuchi M. Moriyama K. (2021). Development of novel bone-like nanocomposite coating of hydroxyapatite/collagen on titanium by modified electrophoretic deposition. J. Biomed. Mater Res. A 109, 1905–1911. 10.1002/jbm.a.37182 33786996

[B49] Ke D. Vu A. A. Bandyopadhyay A. Bose S. (2019). Compositionally graded doped hydroxyapatite coating on titanium using laser and plasma spray deposition for bone implants. Acta Biomater. 84, 414–423. 10.1016/j.actbio.2018.11.041 30500448 PMC6485960

[B50] Kellesarian S. V. Malignaggi V. R. Kellesarian T. V. Bashir Ahmed H. Javed F. (2018). Does incorporating collagen and chondroitin sulfate matrix in implant surfaces enhance osseointegration? A systematic review and meta-analysis. Int. J. Oral Maxillofac. Surg. 47, 241–251. 10.1016/j.ijom.2017.10.010 29096932

[B51] Kim B. S. Kim J. S. Park Y. M. Choi B. Y. Lee J. (2013). Mg ion implantation on SLA-treated titanium surface and its effects on the behavior of mesenchymal stem cell. Mater Sci. Eng. C Mater Biol. Appl. 33, 1554–1560. 10.1016/j.msec.2012.12.061 23827608

[B52] Kim K. H. Ramaswamy N. (2009). Electrochemical surface modification of titanium in dentistry. Dent. Mater J. 28, 20–36. 10.4012/dmj.28.20 19280965

[B53] Kim N. H. Lee S. H. Ryu J. J. Choi K. H. Huh J. B. (2015). Effects of rhBMP-2 on sandblasted and acid etched titanium implant surfaces on bone regeneration and osseointegration: spilt-mouth designed pilot study. Biomed. Res. Int. 2015, 1–11. 10.1155/2015/459393 PMC4609358 26504807

[B54] Kylmäoja E. Holopainen J. Abushahba F. Ritala M. Tuukkanen J. (2022). Osteoblast attachment on titanium coated with hydroxyapatite by atomic layer deposition. Biomolecules 12, 654. 10.3390/biom12050654 35625580 PMC9138598

[B55] Le Guéhennec L. Soueidan A. Layrolle P. Amouriq Y. (2007). Surface treatments of titanium dental implants for rapid osseointegration. Dent. Mater 23, 844–854. 10.1016/j.dental.2006.06.025 16904738

[B56] Leedy M. R. Jennings J. A. Haggard W. O. Bumgardner J. D. (2014). Effects of VEGF-loaded chitosan coatings. J. Biomed. Mater Res. A 102, 752–759. 10.1002/jbm.a.34745 23564543

[B57] Li G. Zheng T. Wu L. Han Q. Lei Y. Xue L. (2021). Bionic microenvironment-inspired synergistic effect of anisotropic micro-nanocomposite topology and biology cues on peripheral nerve regeneration. Sci. Adv. 7, eabi5812. 10.1126/sciadv.abi5812 34233882 PMC8262819

[B58] Li X. Li Y. Liao Y. Li J. Zhang L. Hu J. (2014). The effect of magnesium-incorporated hydroxyapatite coating on titanium implant fixation in ovariectomized rats. Int. J. Oral Maxillofac. Implants 29, 196–202. 10.11607/jomi.2893 24451871

[B59] Li Z. Y. Lam W. M. Yang C. Xu B. Ni G. X. Abbah S. A. (2007). Chemical composition, crystal size and lattice structural changes after incorporation of strontium into biomimetic apatite. Biomaterials 28, 1452–1460. 10.1016/j.biomaterials.2006.11.001 17140655

[B60] Liu C. F. Chang K. C. Sun Y. S. Nguyen D. T. Huang H. H. (2021). Combining sandblasting, alkaline etching, and collagen immobilization to promote cell growth on biomedical titanium implants. Polym. (Basel) 13, 2550. 10.3390/polym13152550 PMC8347351 34372152

[B61] Liu Y. Enggist L. Kuffer A. F. Buser D. Hunziker E. B. (2007). The influence of BMP-2 and its mode of delivery on the osteoconductivity of implant surfaces during the early phase of osseointegration. Biomaterials 28, 2677–2686. 10.1016/j.biomaterials.2007.02.003 17321590

[B62] Liu Y. Luo D. Wang T. (2016). Hierarchical structures of bone and bioinspired bone tissue engineering. Small 12, 4611–4632. 10.1002/smll.201600626 27322951

[B63] López-Valverde N. Flores-Fraile J. Ramírez J. M. Sousa B. M. Herrero-Hernández S. López-Valverde A. (2020). Bioactive surfaces vs. Conventional surfaces in titanium dental implants: a comparative systematic review. J. Clin. Med. 9, 2047. 10.3390/jcm9072047 32610687 PMC7408888

[B64] Lupi S. M. Rodriguez Y. B. A. Cassinelli C. Iviglia G. Tallarico M. Morra M. (2019). Covalently-linked hyaluronan versus acid etched titanium dental implants: a crossover rct in humans. Int. J. Mol. Sci. 20, 763. 10.3390/ijms20030763 30754668 PMC6387289

[B65] Lupi S. M. Torchia M. Rizzo S. (2021). Biochemical modification of titanium oral implants: evidence from *in vivo* studies. Mater. (Basel) 14, 2798. 10.3390/ma14112798 PMC8197372 34074006

[B66] Ma Q. L. Fang L. Jiang N. Zhang L. Wang Y. Zhang Y. M. (2018). Bone mesenchymal stem cell secretion of sRANKL/OPG/M-CSF in response to macrophage-mediated inflammatory response influences osteogenesis on nanostructured Ti surfaces. Biomaterials 154, 234–247. 10.1016/j.biomaterials.2017.11.003 29144982

[B67] Ma Q.-L. Zhao L.-Z. Liu R.-R. Jin B.-Q. Song W. Wang Y. (2014). Improved implant osseointegration of a nanostructured titanium surface via mediation of macrophage polarization. Biomaterials 35, 9853–9867. 10.1016/j.biomaterials.2014.08.025 25201737

[B68] Ma T. Wang C. X. Ge X. Y. Zhang Y. (2023). Applications of polydopamine in implant surface modification. Macromol. Biosci. 23, e2300067. 10.1002/mabi.202300067 37229654

[B69] Marín-Pareja N. Cantini M. González-García C. Salvagni E. Salmerón-Sánchez M. Ginebra M. P. (2015). Different organization of type I collagen immobilized on silanized and nonsilanized titanium surfaces affects fibroblast adhesion and fibronectin secretion. ACS Appl. Mater Interfaces 7, 20667–20677. 10.1021/acsami.5b05420 26322620

[B70] Nagayasu-Tanaka T. Nozaki T. Miki K. Sawada K. Kitamura M. Murakami S. (2017). FGF-2 promotes initial osseointegration and enhances stability of implants with low primary stability. Clin. Oral Implants Res. 28, 291–297. 10.1111/clr.12797 26919334 PMC5347960

[B71] Panzavolta S. Torricelli P. Casolari S. Parrilli A. Fini M. Bigi A. (2018). Strontium-Substituted hydroxyapatite-gelatin biomimetic scaffolds modulate bone cell response. Macromol. Biosci. 18, e1800096. 10.1002/mabi.201800096 29877029

[B72] Park J. Y. Davies J. E. (2000). Red blood cell and platelet interactions with titanium implant surfaces. Clin. Oral Implants Res. 11, 530–539. 10.1034/j.1600-0501.2000.011006530.x 11168246

[B73] Patty D. J. Nugraheni A. D. Dewi Ana I. Yusuf Y. (2022). Mechanical characteristics and bioactivity of nanocomposite hydroxyapatite/collagen coated titanium for bone tissue engineering. Bioeng. (Basel) 9, 784. 10.3390/bioengineering9120784 PMC9774233 36550990

[B74] Prezas P. R. Soares M. J. Borges J. P. Silva J. C. Oliveira F. J. Graça M. P. F. (2023). Bioactivity enhancement of plasma-sprayed hydroxyapatite coatings through non-contact corona electrical charging. Nanomater. (Basel) 13, 1058. 10.3390/nano13061058 PMC10058569 36985952

[B75] Qi H. Shi M. Ni Y. Mo W. Zhang P. Jiang S. (2021). Size-confined effects of nanostructures on fibronectin-induced macrophage inflammation on titanium implants. Adv. Healthc. Mater. 10, 2100994. 10.1002/adhm.202100994 34196125

[B76] Raju K. Mani U. M. Vaidyanathan A. K. (2023). Evaluating the osteogenic potential of insulin-like growth factor-1 microspheres on osteoblastic activity around dental implants in patients with type 2 diabetes mellitus using bone scintigraphy: a split-mouth randomized controlled trial. J. Prosthet. Dent. 129, 561–565. 10.1016/j.prosdent.2021.06.016 34294423

[B77] Ramazanoglu M. Lutz R. Ergun C. Von Wilmowsky C. Nkenke E. Schlegel K. A. (2011). The effect of combined delivery of recombinant human bone morphogenetic protein-2 and recombinant human vascular endothelial growth factor 165 from biomimetic calcium-phosphate-coated implants on osseointegration. Clin. Oral Implants Res. 22, 1433–1439. 10.1111/j.1600-0501.2010.02133.x 21418332

[B78] Sartori M. Giavaresi G. Parrilli A. Ferrari A. Aldini N. N. Morra M. (2015). Collagen type I coating stimulates bone regeneration and osteointegration of titanium implants in the osteopenic rat. Int. Orthop. 39, 2041–2052. 10.1007/s00264-015-2926-0 26206261

[B79] Scarano A. Lorusso F. Orsini T. Morra M. Iviglia G. Valbonetti L. (2019). Biomimetic surfaces coated with covalently immobilized collagen type I: an X-ray photoelectron spectroscopy, atomic force microscopy, micro-CT and histomorphometrical study in rabbits. Int. J. Mol. Sci. 20, 724. 10.3390/ijms20030724 30744023 PMC6387268

[B80] Schliephake H. Rublack J. Förster A. Schwenzer B. Reichert J. Scharnweber D. (2015). Functionalization of titanium implants using a modular system for binding and release of VEGF enhances bone-implant contact in a rodent model. J. Clin. Periodontol. 42, 302–310. 10.1111/jcpe.12370 25640057

[B81] Schliephake H. Strecker N. Förster A. Schwenzer B. Reichert J. Scharnweber D. (2012). Angiogenic functionalisation of titanium surfaces using nano-anchored VEGF - an *in vitro* study. Eur. Cell Mater 23, 161–169. discussion 169. 10.22203/ecm.v023a12 22415802

[B82] Schopper C. Moser D. Goriwoda W. Ziya-Ghazvini F. Spassova E. Lagogiannis G. (2005). The effect of three different calcium phosphate implant coatings on bone deposition and coating resorption: a long-term histological study in sheep. Clin. Oral Implants Res. 16, 357–368. 10.1111/j.1600-0501.2004.01080.x 15877757

[B83] Schulz M. C. Korn P. Stadlinger B. Range U. Möller S. Becher J. (2014). Coating with artificial matrices from collagen and sulfated hyaluronan influences the osseointegration of dental implants. J. Mater Sci. Mater Med. 25, 247–258. 10.1007/s10856-013-5066-3 24113890

[B84] Shao J. Weng L. Li J. Lin H. Wang H. Lin J. (2022). Regulation of macrophage polarization by mineralized collagen coating to accelerate the osteogenic differentiation of mesenchymal stem cells. ACS Biomater. Sci. Eng. 8, 610–619. 10.1021/acsbiomaterials.1c00834 34991308

[B85] Smeets R. Stadlinger B. Schwarz F. Beck-Broichsitter B. Jung O. Precht C. (2016). Impact of dental implant surface modifications on osseointegration. Biomed. Res. Int. 2016, 1–16. 10.1155/2016/6285620 PMC4958483 27478833

[B86] Souza J. C. M. Sordi M. B. Kanazawa M. Ravindran S. Henriques B. Silva F. S. (2019). Nano-scale modification of titanium implant surfaces to enhance osseointegration. Acta Biomater. 94, 112–131. 10.1016/j.actbio.2019.05.045 31128320

[B87] Stadlinger B. Hintze V. Bierbaum S. Möller S. Schulz M. C. Mai R. (2012). Biological functionalization of dental implants with collagen and glycosaminoglycans-A comparative study. J. Biomed. Mater Res. B Appl. Biomater. 100, 331–341. 10.1002/jbm.b.31953 22102613

[B88] Sul Y. T. Johansson C. B. Röser K. Albrektsson T. (2002). Qualitative and quantitative observations of bone tissue reactions to anodised implants. Biomaterials 23, 1809–1817. 10.1016/s0142-9612(01)00307-6 11950051

[B89] Tabrizi R. Sadeghi H. M. Ghasemi K. Khayati A. Jafarian M. (2022). Does biphasic calcium phosphate-coated surface increase the secondary stability in dental implants? A split-mouth study. J. Maxillofac. Oral Surg. 21, 557–561. 10.1007/s12663-020-01448-2 35712432 PMC9192867

[B90] Tomasi C. Derks J. (2022). Etiology, occurrence, and consequences of implant loss. Periodontol. 2000 88, 13–35. 10.1111/prd.12408 35103324 PMC9306999

[B91] Tredwin C. J. Georgiou G. Kim H.-W. Knowles J. C. (2013). Hydroxyapatite, fluor-hydroxyapatite and fluorapatite produced via the sol–gel method: bonding to titanium and scanning electron microscopy. Dent. Mater. 29, 521–529. 10.1016/j.dental.2013.02.002 23518245

[B92] Trisi P. Lazzara R. Rebaudi A. Rao W. Testori T. Porter S. S. (2003). Bone-implant contact on machined and dual acid-etched surfaces after 2 months of healing in the human maxilla. J. Periodontol. 74, 945–956. 10.1902/jop.2003.74.7.945 12931756

[B93] Wang J. Yang B. Guo S. Yu S. Li H. (2023). Manufacture of titanium alloy materials with bioactive sandblasted surfaces and evaluation of osseointegration properties. Front. Bioeng. Biotechnol. 11, 1251947. 10.3389/fbioe.2023.1251947 37671189 PMC10475539

[B94] Wang J. Zhao Q. Fu L. Zheng S. Wang C. Han L. (2022). CD301b+ macrophages mediate angiogenesis of calcium phosphate bioceramics by CaN/NFATc1/VEGF axis. Bioact. Mater. 15, 446–455. 10.1016/j.bioactmat.2022.02.004 35386349 PMC8958385

[B95] Wang X. Xu S. Zhou S. Xu W. Leary M. Choong P. (2016). Topological design and additive manufacturing of porous metals for bone scaffolds and orthopaedic implants: a review. Biomaterials 83, 127–141. 10.1016/j.biomaterials.2016.01.012 26773669

[B96] Xu Y. Li H. Wu J. Yang Q. Jiang D. Qiao B. (2018). Polydopamine-induced hydroxyapatite coating facilitates hydroxyapatite/polyamide 66 implant osteogenesis: an *in vitro* and *in vivo* evaluation. Int. J. Nanomedicine 13, 8179–8193. 10.2147/ijn.s181137 30555233 PMC6280913

[B97] Yang D. H. Lee D. W. Kwon Y. D. Kim H. J. Chun H. J. Jang J. W. (2015). Surface modification of titanium with hydroxyapatite-heparin-BMP-2 enhances the efficacy of bone formation and osseointegration *in vitro* and *in vivo* . J. Tissue Eng. Regen. Med. 9, 1067–1077. 10.1002/term.1973 25524250

[B98] Yasunaga M. Kobayashi F. Sogo Y. Murotomi K. Hirose M. Hara Y. (2022). The enhancing effects of heparin on the biological activity of FGF-2 in heparin-FGF-2-calcium phosphate composite layers. Acta Biomater. 148, 345–354. 10.1016/j.actbio.2022.06.013 35697197

[B99] Yeo I. S. Min S. K. Kang H. K. Kwon T. K. Jung S. Y. Min B. M. (2015). Identification of a bioactive core sequence from human laminin and its applicability to tissue engineering. Biomaterials 73, 96–109. 10.1016/j.biomaterials.2015.09.004 26406450

[B100] Zhao B. Li X. Xu H. Jiang Y. Wang D. Liu R. (2020). Influence of simvastatin-strontium-hydroxyapatite coated implant formed by micro-arc oxidation and immersion method on osteointegration in osteoporotic rabbits. Int. J. Nanomedicine 15, 1797–1807. 10.2147/ijn.s244815 32214812 PMC7083628

[B101] Zhao S. F. Jiang Q. H. Peel S. Wang X. X. He F. M. (2013). Effects of magnesium-substituted nanohydroxyapatite coating on implant osseointegration. Clin. Oral Implants Res. 24 (Suppl. A100), 34–41. 10.1111/j.1600-0501.2011.02362.x 22145854

[B102] Zhao Y. Bai L. Zhang Y. Yao R. Sun Y. Hang R. (2022). Type I collagen decorated nanoporous network on titanium implant surface promotes osseointegration through mediating immunomodulation, angiogenesis, and osteogenesis. Biomaterials 288, 121684. 10.1016/j.biomaterials.2022.121684 35995624

[B103] Zhe W. Dong C. Sefei Y. Dawei Z. Kui X. Xiaogang L. (2016). Facile incorporation of hydroxyapatite onto an anodized Ti surface via a mussel inspired polydopamine coating. Appl. Surf. Sci. 378, 496–503. 10.1016/j.apsusc.2016.03.094

